# Hospital Records and Medical Research

**Published:** 1904-12-17

**Authors:** 


### Hospital Records and Medical Research.

It may be regarded as a great defect in the medical literature of to day that so large a proportion of the papers which appear in the various periodicals are compounded from previous publications instead of being based, as they should be, upon personal observations and, if these are not sufficiently extensive, upon the official records of the hospitals. One result of the present custom is the over-rating of medical and surgical therapeutic measures. In these utilitarian times a man is thought to be foolish if he makes public his failures, and usually he hesitates to do so, although failures are, as a rule, more instructive than successes as guides to future usage. What the student requires is not an *ex parte* statement in which the triumphs of medicine and surgery are set forth alone; he needs to know also the cases which have ended in failure. It is manifestly desirable, therefore, since the personal experiences of any individual are usually too limited to justify generalisations being made from them, not to mention the inevitable drawbacks of personal factors and tendency to bias, that every hospital should preserve careful clinical records, including actual morbid material such as microscope specimens and so forth, and should keep all this valuable material in a condition to be readily accessible for reference. That these remarks are called for will be apparent to anyone who surveys the current numbers of the medical journals. On turning over the pages of one journal after another the same feature stands sharply out—namely, that a great deal of the matter is based on the printed literature, instead of being the outcome of personal observations or of original research among the official records of institutions. The consequence is that an error once made is liable to be perpetuated year after year in the text-books and periodicals until it has become so firmly rooted in the medical mind that a great effort is required to efface the mistaken doctrine within a reasonable time. To quote only one instance. For years it has been the habit of English obstetricians to teach that plugging the vagina for accidental hæmorrhage in the later months of pregnancy is one of the most unpardonable sins. Now, however, a great authority comes forward to say that he has habitually practised the condemned method with results very much better than those which follow the procedure which has been so strenuously upheld in the English Medical Schools. If this statement be true, and there is not the slightest reason to doubt its correctness, it is clear that those who have for years taken special trouble to condemn plugging the vagina as a means of treating accidental hæmorrhage must have based their opinions on entirely fallacious premisses that is to say, they have accepted as their instructor the text-book in place of practical experience. No doubt it is very much easier to sit in a library and collect for analysis the cases reported by different observers, than it is to compose an article by visiting such hospitals as preserve records and collecting and using the information gained in this way; and the more so as, it must be confessed, facilities for the method here suggested are not always provided, for man[illegible] fair-sized hospitals keep no proper permanen[illegible] records at all, and at several of those institution[illegible] where clinical notes are preserved they are no[illegible] indexed, or in other ways rendered suitable fo[illegible] purposes of reference. Every hospital should con[illegible]sider it an essential duty to treasure up the clinica[illegible] experience gained within its walls, and to arrang[illegible] its stores of information in such a way that the[illegible] may most readily be available to the scientifi[illegible] researcher, whose labours have for their ultimate object the welfare of mankind.

